# Morpho-Physiochemical Indices and Transcriptome Analysis Reveal the Role of Glucosinolate and Erucic Acid in Response to Drought Stress during Seed Germination of Rapeseed

**DOI:** 10.3390/ijms25063308

**Published:** 2024-03-14

**Authors:** Xueying Ai, Ali Mahmoud El-Badri, Maria Batool, Hongxiang Lou, Gengdong Gao, Chenyang Bai, Zongkai Wang, Chunji Jiang, Xinhua Zhao, Bo Wang, Jie Kuai, Zhenghua Xu, Jing Wang, Graham John King, Haiqiu Yu, Guangsheng Zhou, Tingdong Fu

**Affiliations:** 1 College of Agronomy, Shenyang Agricultural University, Shenyang 110161, China; aixueying1994@163.com (X.A.); jiangchunji2002@163.com (C.J.); xinhua_zhao@syau.edu.cn (X.Z.); yuhaiqiu@syau.edu.cn (H.Y.); zhougs@mail.hzau.edu.cn (G.Z.); 2 MOA Key Laboratory of Crop Ecophysiology and Farming System in the Middle Reaches of the Yangtze River, College of Plant Science & Technology, Huazhong Agricultural University, Wuhan 430070, China; alyelbadry@webmail.hzau.edu.cn (A.M.E.-B.); maria.batool@webmail.hzau.edu.cn (M.B.); davidlou_2013@163.com (H.L.); ggd@webmail.hzau.edu.cn (G.G.); baicy@webmail.hzau.edu.cn (C.B.); wangzongkai@webmail.hzau.edu.cn (Z.W.); wangbo@mail.hzau.edu.cn (B.W.); kuaijie@mail.hzau.edu.cn (J.K.); xzh@mail.hzau.edu.cn (Z.X.); rapelab@mail.hzau.edu.cn (T.F.); 3 Field Crops Research Institute, Agricultural Research Center (ARC), Giza 12619, Egypt; 4 Southern Cross Plant Science, Southern Cross University, Lismore, NSW 2480, Australia; graham.king@scu.edu.au

**Keywords:** rapeseed, germination, glucosinolate, erucic acid, transcriptome, seed priming

## Abstract

The global expansion of rapeseed seed quality has been focused on maintaining glucosinolate (GSL) and erucic acid (EA) contents. However, the influence of seed GSL and EA contents on the germination process under drought stress remains poorly understood. Herein, 114 rapeseed accessions were divided into four groups based on GSL and EA contents to investigate their performance during seed imbibition under drought stress. Our results revealed significant variations in seed germination-related traits, particularly with higher GSL and EA, which exhibited higher germination % (G%) and lower mean germination time (MGT) under drought stress conditions. Moreover, osmoregulation, enzymatic system and hormonal regulation were improved in high GSL and high EA (HGHE) versus low GSL and low EA (LGLE) seeds, indicating the essential protective role of GSL and EA during the germination process in response to drought stress. The transcriptional regulation mechanism for coordinating GSL–EA-related pathways in response to drought stress during seed imbibition was found to involve the differential expression of sugar metabolism-, antioxidant-, and hormone-related genes with higher enrichment in HGHE compared to LGLE seeds. GO enrichment analysis showed higher variations in transcription regulator activity and DNA-binding transcription factors, as well as ATP and microtubule motor activity in GSL–EA-related pathways. Furthermore, KEGG analysis identified cellular processes, environmental information processing, and metabolism categories, with varied gene participation between GSL, EA and GSL–EA-related pathways. For further clarification, QY7 (LGLE) seeds were primed with different concentrations of GSL and EA under drought stress conditions. The results showed that 200 μmol/L of GSL and 400 μmol/L of EA significantly improved G%, MGT, and seedling fresh weight, besides regulating stress and fatty acid responsive genes during the seed germination process under drought stress conditions. Conclusively, exogenous application of GSL and EA is considered a promising method for enhancing the drought tolerance of LGLE seeds. Furthermore, the current investigation could provide a theoretical basis of GSL and EA roles and their underlying mechanisms in stress tolerance during the germination process.

## 1. Introduction

Rapeseed (*Brassica napus* L.) is the second largest oilseed producer worldwide, accounting for approximately 12% of global vegetable oil production [1]. It serves as a source of cooking oil for humans, protein-rich animal feed due to its ideal amino acid, higher fiber, essential vitamins and vital minerals contents, as well as renewable materials for industries [2]. Moreover, rapeseed occupies approximately 7 million hectares of land, which accounts for about 90% of rapeseed production in China, especially in the Yangtze River Basin region [3].

With global climate changes, drought is a critical environmental factor that affects crop yields, and it is intensifying in certain regions [4,5,6]. Currently, approximately 41.4% of the global land area commonly experiences drought stress, leading to an annual yield reduction of 21–30% [7,8]. Moreover, drought tolerance is a complicated process due to its variation with stress duration and intensity relative to plant growth and development [9]. On the other side, drought stress adversely affects rapeseed at all growth stages (seed to seed), which reduces the germination and seedling growth with oxidative damages and an impaired antioxidant defense system [10]; thereby, tolerant rapeseed cultivar development is a critical goal for breeders.

Seed germination is a crucial stage in crop production and is significantly affected by water availability, which substantially affects the germination speed and rate [11]. Furthermore, intrinsic factors, such as seed quality (composition of major storage components), have a notable impact on rapeseed seeds [12,13]; therefore, variations in quality considerably influences the resilience of seeds to stress [14]. Moreover, the mobilization of stored protein and lipids in seeds facilitates physiological metabolism during the germination process, resulting in a gradual decrease in primary reserves [15]. Protein translation ability is significantly associated with seed viability, as the inhibition of protein translation results in decreased seed germination [16,17]. A previous study reported that enhanced protein metabolism contributed to increased germination vigor in high-oil rapeseed seeds [18]. In addition, glycolysis plays a vital role in sugar mobilization, providing energy and increased metabolites required for germination and seedling growth, and it is closely correlated with germination speed and seedling quality under drought stress conditions [19,20]. Sugars and proteins are involved in energy metabolism, playing an important role in osmoregulation, which is crucial for mitigating reactive oxygen species (ROS) during seed germination under drought stress [21]. Moreover, these compounds facilitate cellular water uptake by reducing the osmotic potential of cells and ensuring intracellular homeostasis [22].

Under drought stress conditions, cultivars with a faster germination rate (GR) and higher germination percentage are associated with enhanced cellular osmoregulation and antioxidant capabilities [23]. Over the past decade, recurring autumn and winter droughts have compromised the consistency of mature rapeseed seedlings, inhibited the achievement of optimal planting densities, and significantly reduced production efficiency [1]. In particular, increased activity of antioxidant enzymes in seed radicles is fundamental for drought resilience in plants [24,25]. Furthermore, plant hormones play an important role in breaking seed dormancy, enhancing germination, and modulating physiological responses under stress conditions [26]. Gibberellic acid (GA) and abscisic acid (ABA) are the primary hormones that regulate seed germination, especially with a higher GA/ABA ratio [27]. Additionally, ABA stimulates osmoregulatory substance accumulation, including soluble sugars, proteins, and amino acids, that regulate cellular osmotic balance under stress conditions [28].

The global expansion of rapeseed seed quality has been dependent on maintaining the levels of seed glucosinolate (GSL) and erucic acid (EA) [29]. GSL (sugar derivative) is a crucial secondary metabolite in the plant response to stress that modulates cellular osmoregulation during seed germination, especially under drought stress conditions [30]. Its decomposition products (isothiocyanates, nitriles, and thiocyanates) participate in various defense mechanisms [31,32]. Moreover, plants increase GSL levels and their transportation through sulfur assimilation pathways and amino acid metabolism, which modify the osmotic potential and water uptake in response to drought stress [33]. Variation in the fatty acid (FA) composition of seeds is essential for plant responses to environmental stresses, in particular, EA (long-chain polyunsaturated FA) exhibited a markedly affected GR under stress conditions in rapeseed [34]. During seed imbibition, membrane lipids are repaired and converted to a normal bilayer; nevertheless, abiotic stress can hamper membrane remodeling that affects seed germination [19]. However, the relationship between seed composition, drought tolerance, and rapeseed quality improvement is poorly understood.

This study is based on the recognition of the high economic value and multifunctionality of rapeseed, as well as the significant impact of drought on rapeseed and the relation of germination with GSL- and EA-related metabolism. The aim was to discern the underlying mechanisms driving the disparities in drought tolerance during the germination stage among different types of rapeseed seeds. Herein, 114 rapeseed accessions with different levels of GSL and EA were subjected to PEG-induced drought stress during the seed germination stage. Morpho-physiochemical indices were determined at seed imbibition of the four rapeseed accession groups, categorized based on their GSL and EA contents. Additionally, transcriptomic analysis was conducted to investigate the molecular pathways involved in GSL- and EA-related metabolism. Exogenously applied GSL and EA via seed priming could improve the adaptability of rapeseed to drought stress at the seed germination stage. This study provides a theoretical foundation and practical guidance of the relation between rapeseed seed quality and drought tolerance.

## 2. Results

### 2.1. Variations in the Germination Rates of Various Accessions under Drought Stress

**Figure 1:**
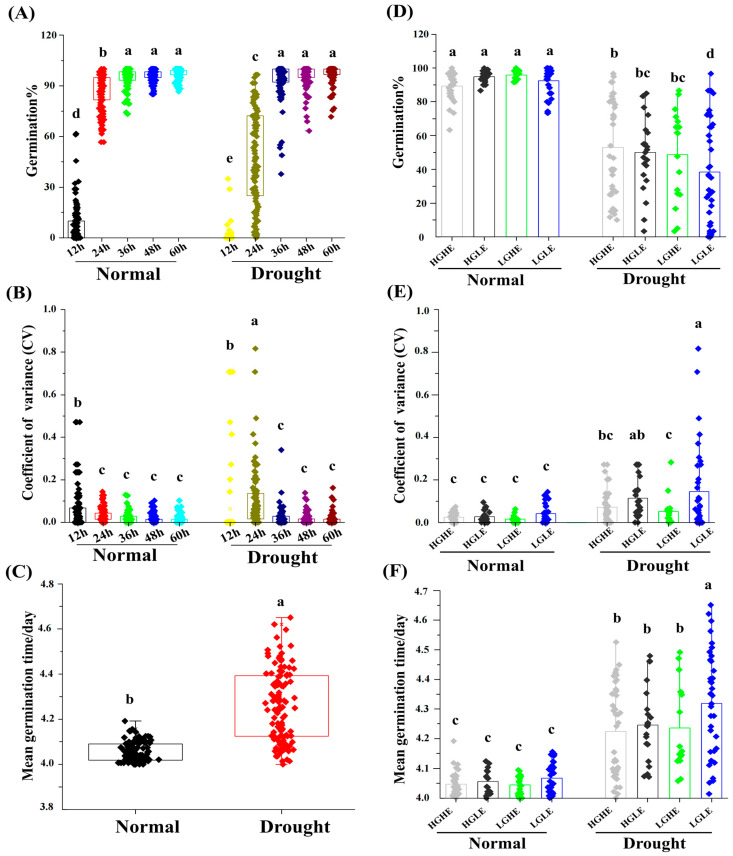
Variation effect of 114 rapeseed accessions at 12, 24, 36, 48 and 60 h of seed imbibition in terms of (**A**) germination%, (**B**) coefficient of variance (CV) and (**C**) mean germination time/day (MGT) under normal and drought stress conditions. Variation among the four seed type groups at 24 h in terms of (**D**) germination%, (**E**) coefficient of variance (CV) and (**F**) mean germination time/day (MGT) under normal and drought stress conditions. The different letters indicate significant differences at *p* < 0.05 using Duncan’s multiple range tests.

To investigate the differences in seed germination among different quality types of rapeseed seeds under drought stress conditions, a total of 114 representative accessions were selected, encompassing a wide range of rapeseed cultivars and research lines. G%, CV, and MGT were calculated at five time points (12, 24, 36, 48, and 60 h) during germination under treatment conditions (Figure 1A–F). The average G% values at the five time points were recorded as 6.91, 86.71, 94.94, 96.26, and 97.03% with corresponding CV values of 0.06, 0.03, 0.02, 0.02, and 0.01, respectively, under normal conditions of 114 accessions. Meanwhile, under drought stress conditions, it was recorded as 0.94, 47.01, 93.65, 96.06 and 97.04% with respective CV values of 0.06, 0.10, 0.02, 0.02, and 0.01, respectively (Figure 1A,B). Moreover, the average MGT of all the studied accessions was calculated as 4.05 and 4.26 days under the normal and drought stress conditions, respectively (Figure 1C).

Based on the aforementioned results, the 24 h time point presented a significant difference in G% between normal and stress conditions in four groups (HGHE, HGLE, LGHE, and LGLE) of rapeseed accessions. Therefore, 24 h was chosen as the critical time point to evaluate the rapeseed seed response to drought stress (Figure 1D–F). Moreover, the average decrease in G% was recorded as 40.77, 47.27, 49.17, and 58.49% for HGHE, HGLE, LGHE, and LGLE groups, respectively, under drought stress versus normal conditions, while there were non-significant differences under normal conditions among the four groups (Figure 1D). Additionally, our results indicated significant differences in the average CV of G% and MGT between normal and drought stress conditions among the four groups. The LGLE group showed the highest variation with a CV of 0.15 and an MGT of 4.32 days (Figure 1E,F). Correlation analysis between the relative G% at 24 h, relative MGT, and seed components (PC, OC, EAC, GSLC, LNAC, LAC, and OAC) revealed that both relative germination and MGT under drought stress conditions exhibited significant positive correlations with GSL and EA contents (Table S3). Conclusively, HGHE showed higher G% and shorter MGT versus other groups, especially LGLE, suggesting that GSL and EA contents in rapeseed seeds play a vital role in the seed germination process under drought stress conditions.

### 2.2. Alteration of Phenotype, Osmolytes, and Glucose Metabolism-Related Enzymes in Different Types of Rapeseed Seeds under Drought Stress Conditions

**Figure 2:**
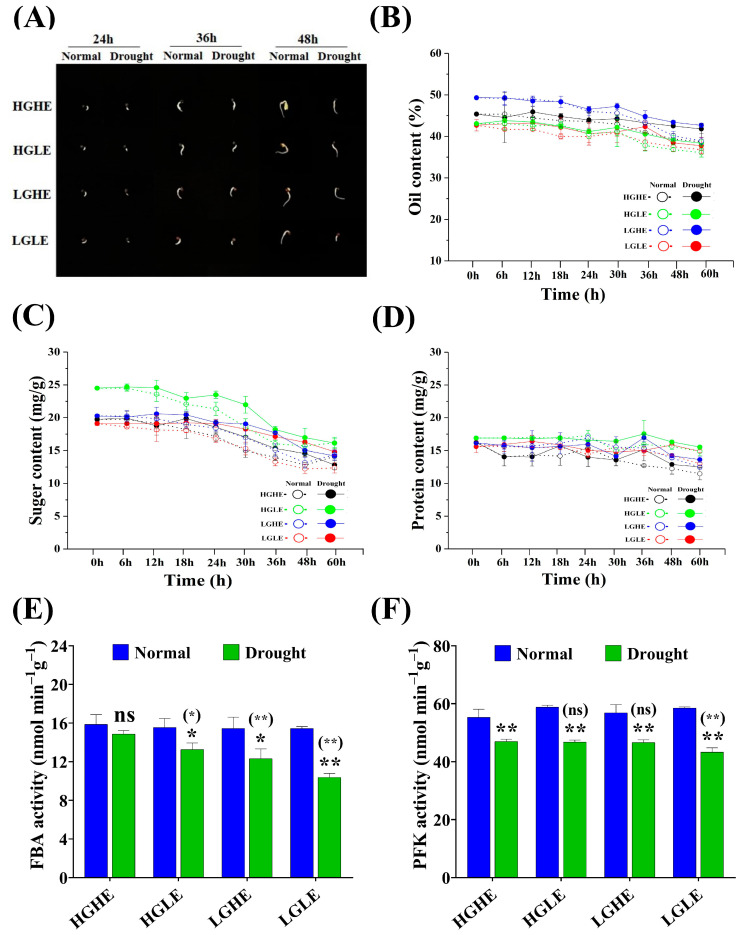
The effect of drought stress on (**A**) seed germination phenomena, (**B**) oil content, (**C**) total soluble sugar content, (**D**) total soluble protein content, (**E**) fructose-bisphosphate aldolase activity, and (**F**) phosphofructokinase activity during the seed germination in four seed type groups of rapeseed. Bars represent ±SE of three replicates. Asterisks indicate significant differences between drought and normal conditions, while asterisks with parentheses indicate significant differences between HGHE and the other three groups under drought conditions (ns: non-significant, * *p* < 0.05, ** *p* < 0.01; Student’s *t*-test).

Based on the analysis of germination-related traits at different time points of the four accession groups, Caoyou2, 7191, Cubs root, and Qinyou7 were selected to represent HGHE, HGLE, LGHE, and LGLE groups, respectively. The germinated seed phenotype of these accessions showed the longest radicles in Caoyou2, followed by 7191, Cubs root, and Qinyou7 at 24, 36, and 48 h under drought stress conditions (Figure 2A). Our results showed that oil content, total soluble sugar, and total soluble protein contents were significantly affected under drought stress conditions in four accessions at different time points, especially at 24 h of seed germination. Moreover, versus normal conditions, oil content, total soluble sugar, and total soluble protein contents were decreased by 0.77, 1.57, 1.08, and 1.62% (oil content); 7.52, 9.08, 6.47, and 12.32% (total soluble sugar); and 9.88, 4.43, 6.11, and 4.67% (total soluble protein) in Caoyou2, 7191, Cubs root, and Qinyou7, respectively, under drought stress conditions. Moreover, the total soluble sugar content was significantly decreased by 35.22 and 34.28% (normal conditions), 26.92 and 14.94% (drought stress conditions) between 0 to 48 h in Caoyou2 and Qinyou7, respectively (Figure 2B–D).

Based on these results, 24 h of seed germination had significant variation among different time points in the four accessions under drought stress conditions. Moreover, FBA and PFK enzyme activity (glucose metabolism) displayed a non-significant variation under normal conditions, while it was higher in Caoyou2 than in the other accessions under drought conditions at 24 h of seed germination. Versus normal conditions, FBA and PFK activity decreased by 1.35, 20.06, 27.46, and 49.76% (FBA) and 6.55, 16.50, 16.68, and 25.15% (PFK) in Caoyou2, 7191, Cubs root, and Qinyou7, respectively, under drought stress conditions (Figure 2E,F). The aforementioned results revealed that GSL and EA play an important role in providing more energy and protection of the cellular component under drought stress conditions by modulating osmolytes and glucose metabolism-related enzymes, especially in the HGHE-type group.

### 2.3. Microstructural-Related Traits and Myrosinase and Lipase Activities Varied in Different Rapeseed Seed Types under Drought Stress Conditions

**Figure 3:**
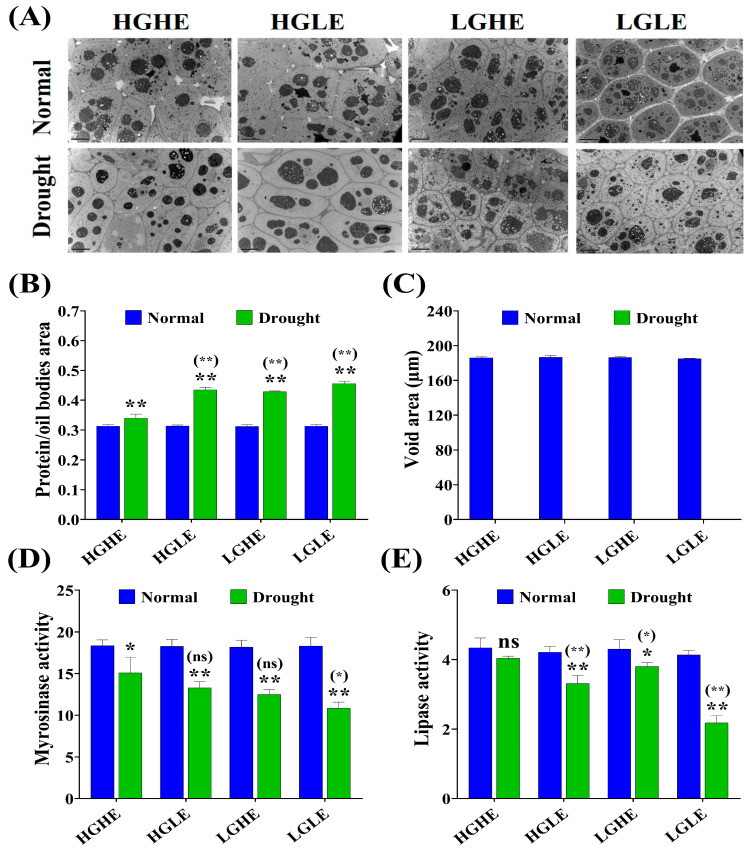
The effect of drought stress on (**A**) seed ultrastructure, (**B**) ratio of protein-to-oil bodies area, (**C**) void area, (**D**) myrosinase activity, and (**E**) lipase activity during the seed germination in four seed type groups of rapeseed. Bars represent ±SE of three replicates. Asterisks indicate significant differences between drought and normal conditions, while asterisks with parentheses indicate significant differences between HGHE and the other three groups under drought conditions (ns: non-significant, * *p* < 0.05, ** *p* < 0.01; Student’s *t*-test).

Under normal conditions, with the decomposition and consumption of protein and oil, cellular void areas became evident at 24 h of seed germination, while it was not observed under drought stress conditions. In addition, drought stress notably enhanced the protein-to-oil body ratio in the four accession types during germination. Protein body/oil area ratio increased by 7.02, 26.58, 25.21, and 31.46% in Caoyou2, 7191, Cubs root, and Qinyou7, respectively, at 24 h under drought stress versus normal conditions. Moreover, a void area between protein and oil bodies was found under normal conditions, while it was absent under drought stress conditions in all studied accessions seeds (Figure 3A–C).

Myrosinase and lipase activities varied significantly between the four accession types at 24 h of seed imbibition, especially under drought stress conditions. Among the four accessions types seeds, the decreased range of those two-enzyme activity was lower in Caoyou2 than in 7191, Cubs root, and Qinyou7, which was increased by 18.64, 38.12, 39.00, and 54.73% (myrosinase) and 8.29, 23.84, 11.12, and 64.08% (lipase), respectively, under drought stress versus normal conditions. Moreover, compared with Caoyou2 (HGHE), the myrosinase activity was decreased by 15.72, 13.38 and 24.03%, while lipase activity was reduced by 10.72, 13.68 and 17.88% in HGLE, LGHE and LGLE, respectively, under drought stress conditions (Figure 3D,E), thus indicating that HGHE had better stress adaptability through GSL- and EA-modulated metabolic activities and microstructure.

### 2.4. Effect of Drought Stress on the Seed’s Defense and Hormonal Systems in Different Types of Rapeseed

**Figure 4:**
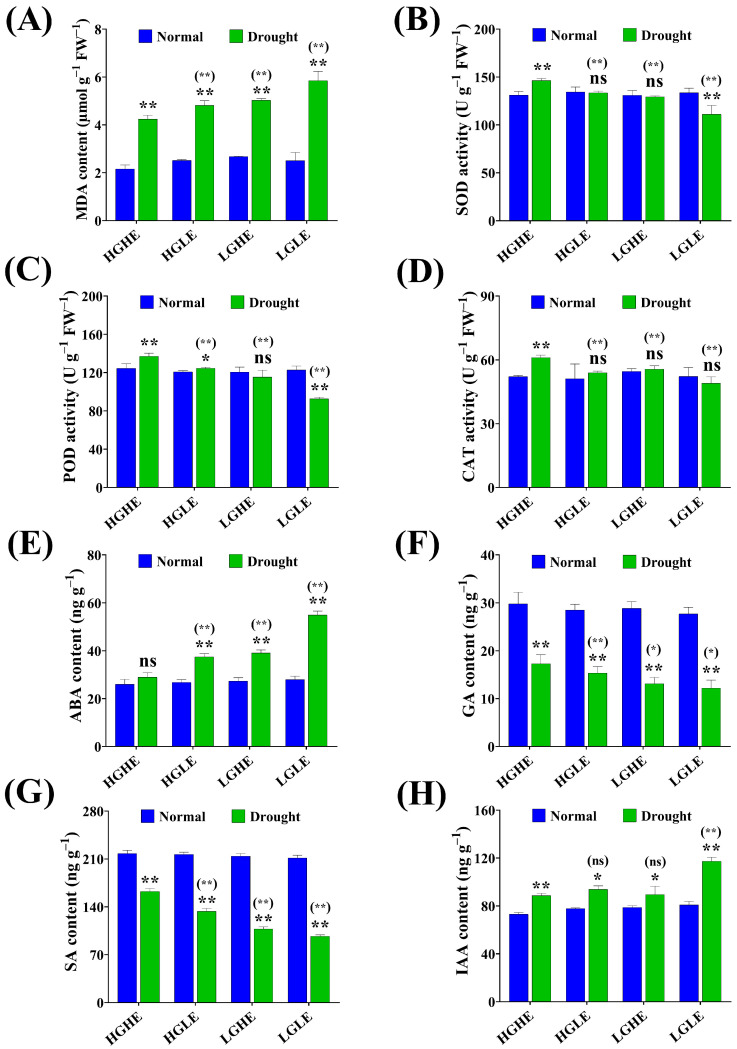
The effect of drought stress on (**A**) malonaldehyde (MDA) content, (**B**) superoxide dismutase activity, (**C**) peroxidase activity, (**D**) catalase activity, (**E**) abscisic acid content, (**F**) gibberellic acid content, (**G**) salicylic acid content, and (**H**) indole acetic acid content during the seed germination in four seed type groups of rapeseed. Bars represent ±SE of three replicates. Asterisks indicate significant differences between drought and normal conditions, while asterisks with parentheses indicate significant differences between HGHE and other three groups under drought conditions (ns: non-significant, * *p* < 0.05, ** *p* < 0.01; Student’s *t*-test).

Drought stress significantly affected the antioxidant capacity, which increased the antioxidant enzyme activity in rapeseed accession seeds except for Qinyou7, resulting in an increase in membrane lipid peroxidation via MDA content (Figure 4A–D). At 24 h of seed imbibition, MDA content was increased by 37.84, 42.00, 43.85, and 46.85% in Caoyou2, 7191, Cubs root and Qinyou7, respectively, which was highest in LGLE-type (Qinyou7) under drought stress versus normal conditions (Figure 4A). On the other side, among the studied types, Caoyou2 (HGHE) exhibited the highest increases by 5.59, 6.31, and 17.98% (SOD); 2.97, 15.18, and 25.26% (POD); and 60, 8.89, and 39.07% (CAT) versus 7191 (HGLE), Cubs root (LGHE) and Qinyou7 (LGLE), respectively, under drought stress conditions (Figure 4B–D). These results suggested that the HGHE-type seeds possessed a higher antioxidant capacity under drought stress conditions.

Under drought stress, different hormonal responses were observed in the studied accessions for stress tolerance, where ABA and IAA levels were increased, while GA and SA levels were significantly decreased in the studied accessions at 24 h of seed imbibition. Compared to Caoyou2 (HGHE), ABA and IAA contents were increased by 47.29, 31.84, and 28.92% (ABA); and 24.46, 20.01, and 23.69% (IAA); while GA and SA contents were decreased by 33.72, 11.41 and 17.88% (GA); and 29.46, 40.38, and 24.03% (SA) in 7191 (HGLE), Cubs root (LGHE) and Qinyou7 (LGLE), respectively, under drought stress conditions (Figure 4E–H). Consequently, the higher content of GSL and EA, especially in HGHE, promoted ROS scavenging through increasing POD and CAT activities to improve seed germination. Besides, HGHE demonstrated a higher GA/ABA ratio and SA content, which was conducive to rapid germination through elevating water absorption and reserve mobilizing enzyme activities under drought stress conditions.

### 2.5. Transcriptome Analysis and Metabolic-Related Pathways in Response to Drought Stress

To investigate the potential molecular mechanism of the four accession groups related to GSL and EA, RNA-seq analysis was carried out using the seeds under normal and drought stress conditions after 24 h of imbibition. Cluster analysis showed that among different types, the number of upregulated genes in HGHE was greater than in LGLE, while it was higher in HGLE versus LGLE and in LGHE versus LGLE (Figure S1A–C).

**Figure 5:**
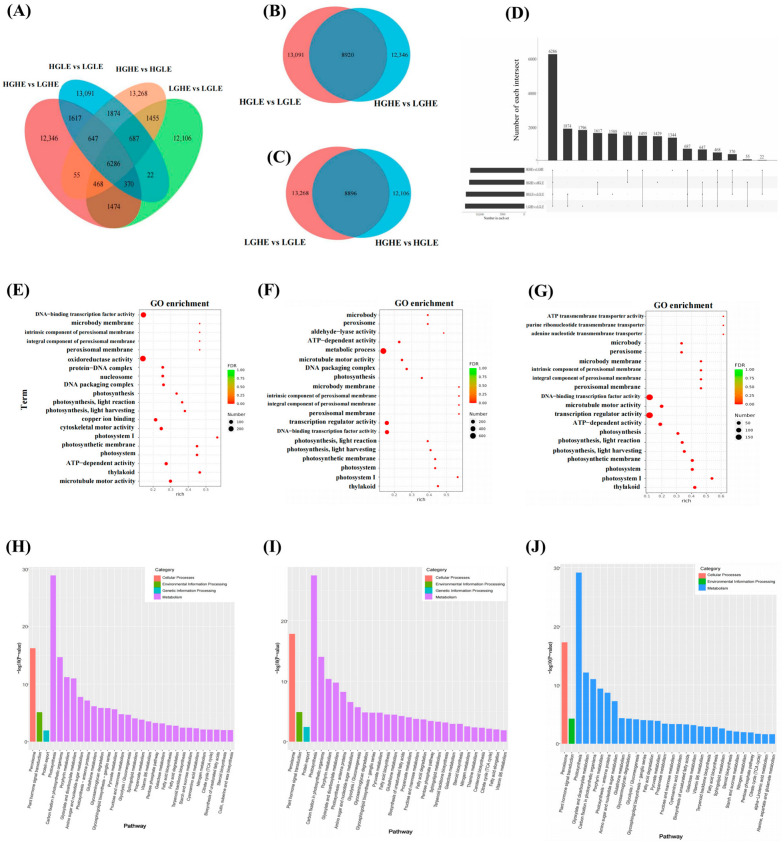
(**A**–**C**) Venn diagrams of the differential gene expression between four groups; (**D**) upset plot of the differential genes related to GSL and EA; (**E**–**G**) gene ontology (GO) annotation analysis of genes related to GSL, EA, and GSL–EA, respectively; and (**H**–**J**) Kyoto Encyclopedia of Genes and Genomes (KEGG) pathway analysis of genes related to GSL, EA, and GSL–EA, respectively.

The analysis identified a total of 6286 differentially expressed genes (DEGs) between HGHE–LGHE and HGLE–LGLE, as well as HGHE–HGLE and LGHE–LGLE, which were commonly associated with GSL and EA (Figure 5A–D). Specifically, there were 12346 DEGs related to GSL (7054 upregulated and 5292 downregulated) in HGHE vs. LGHE, while 13,091 (5403 upregulated DEGs and 7688 downregulated) in HGLE vs. LGLE. Moreover, total 12,106 DEGs related to EA (6490 upregulated and 5616 downregulated) in HGHE vs. HGLE, while 13,268 (6098 upregulated and 7170 downregulated) in LGHE vs. LGLE. Furthermore, 8920 DEGs related to GSL were found in HGLE–LGLE associated with HGHE–LGHE, and 8896 DEGs related to EA were found in LGHE–LGLE associated with HGHE–HGLE (Figure 5A–D and Figure S2A–D).

Moreover, GO and KEGG analyses were performed on GSL, EA and their associated differential genes, where GO enrichment highlighted major variations in DNA-binding transcription factors, seed DNA activity-related pathways, photosynthesis-related pathways, peroxisomes, and ATP activity-related pathways in GSL- and EA-related mechanisms (Figure 5E,F). GO enrichment showed higher variations in transcription regulator activity and DNA-binding transcription factors, followed by ATP- and microtubule motor activity-related pathways in GSL–EA-related pathways (Figure 5G).

Our KEGG analysis identified four categories related to GSL and EA pathways, including cellular processes, environmental information processing, genetic information processing, and metabolism, with differences in gene participation for each category between GSL- and EA-related pathways. Moreover, KEGG analysis emphasized enrichment in peroxisomes, which are essential for enhancing plant sensitivity to ROS under drought stress conditions. In terms of environmental information, there appears to be an emphasis on hormone signaling pathways, crucial for seed germination, while the metabolism category showed differences primarily in photosynthesis, glycolysis, and fatty acid metabolism pathways. Particularly, glycolysis-related pathways were prominent, including the upstream pentose phosphate pathway, glycolysis/gluconeogenesis, and the subsequent citric acid cycle in energy metabolism (Figure 5H,I).

On the other side, the KEGG analysis of associated GSL- and EA-related pathways showed peroxisome enrichment in the cellular processes category, while environmental information focused predominantly on hormonal signaling. In terms of metabolism, variances were primarily in photosynthesis-, glycolysis-, and fatty acid-related metabolism pathways as well as cyanoamino acid, α-linolenic acid, alanine, aspartic acid, and glutamate metabolism. However, protein export was not expressed in GSL–EA-related KEGG pathways, which indicates a distinction between the HGHE and LGLE accessions (Figure 5J).

Comparisons between the accessions in each group were studied to further clarify the GSL and EA metabolic pathways. Differential expression showed 66 genes in HGHE vs. HGLE, 25 genes in HGLE vs. LGLE, 89 genes in HGHE vs. LGHE, and 9 genes in LGHE vs. LGLE (Figure S3A–D). Intersections of HGLE and LGHE with HGHE and LGLE were subjected to GO enrichment analysis for targeted analysis of drought stress responsive genes and seed germination, which was accompanied by variations in organelles and cell membranes as well as changes in enzyme activity. Differential genes of HGHE with HGLE and LGHE, as well as LGLE with HGLE and LGHE, exhibited pronounced peroxidase activity (Figure S3E–H). The KEGG pathway analysis was primarily centered on glycolysis metabolism, and distinct rapeseed seed types demonstrated varied amino acid metabolic patterns (Figure S3I–L). These findings suggested that augmented lipid and glycolysis metabolism, along with enhanced antioxidant defenses, were crucial for the expedited germination rate of the HGHE accession under drought stress conditions.

### 2.6. Gene Expression of Key Metabolic Pathways Associated with Drought Stress

**Figure 6:**
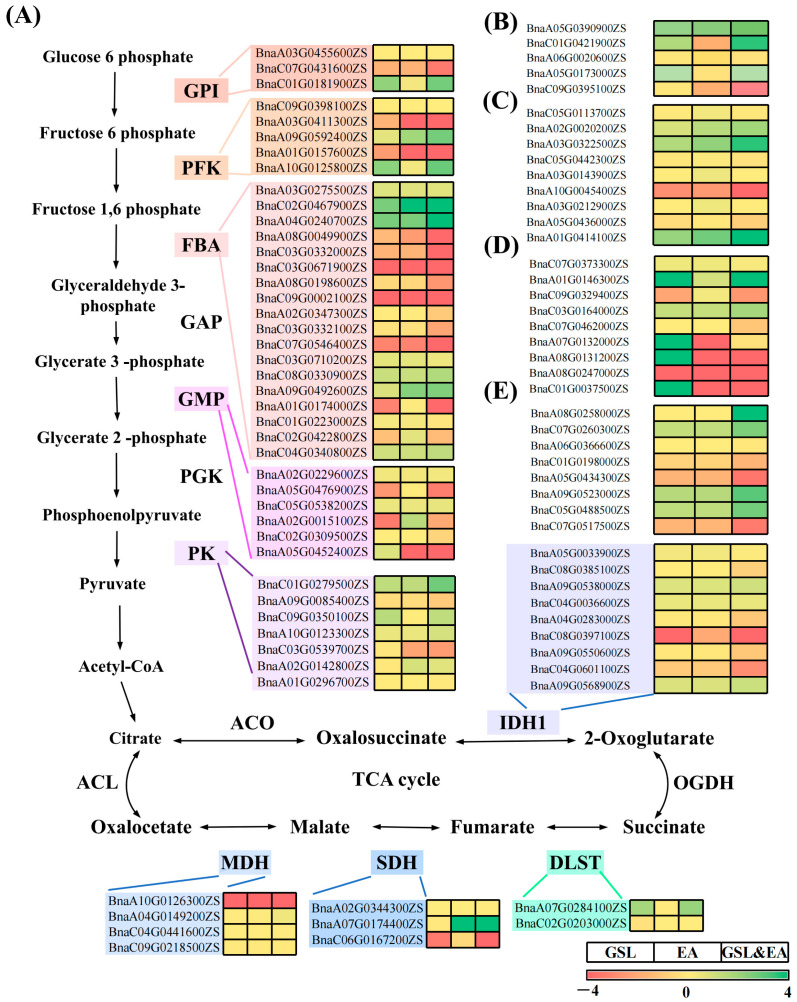
Representative DEGs involved in GSL-, EA-, and GSL–EA-related pathways; (**A**) glucose-; (**B**) myrosinase-; (**C**) lipid-; (**D**) antioxidant-; and (**E**) hormonal metabolism-related genes.

Under drought stress conditions, the HGHE-type seeds exhibited pronounced superiority in the glycolysis process, a critical glucose metabolism pathway during seed germination. This was evident in the activities of GPI, PFK, FBA2, and GMP. In particular, the genes associated with PFK and FBA enzymes underwent significant alterations, with FBA enzyme-related genes being the most differentially expressed, highlighting their pivotal role in seed germination (Figure 6A). Moreover, gene expression levels during the germination processes varied considerably among the seeds with GSL, EA, and GSL–EA contents. The expression levels of *PFK*, *FBA*, *GMP*, and *PK* genes related to GSL and EA were inconsistent. The HGHE-type seeds showed a higher association with key glycolytic-related genes under drought stress conditions. In addition, drought stress affected the expression level of genes involved in the citric acid cycle (a key metabolic pathway) in different accession types. Furthermore, under drought stress, nine CS proteins and three SDH proteins were actively involved in the citric acid cycle (upregulated genes), facilitating the conversion of isocitric acid to ketoglutaric acid, succinic acid to fumarate, and malic acid to oxaloacetic acid. The trend for MDH, involved in the conversion of malic acid to oxaloacetic acid, was unclear (Figure 6A).

In the metabolic pathway of GSL degradation, key genes regulating myrosinase showed an upregulation with three different types (GSL, EA, and GSL–EA) under drought stress conditions (Figure 6B). In the lipid metabolism pathway, genes involved in fatty acid desaturation exhibited upregulation for GSL–EA-related pathways under drought stress conditions; whereas the expression of genes was higher in GSL–EA- than GSL- and EA-related pathways (Figure 6C). Drought stress affected the expression of ROS oxidative metabolism-related genes in seed germination with varying GSL and EA contents. The upregulation of POD- and CAT-related genes was evident during seed germination of HGHE than HGLE and LGHE accessions (Figure 6D). Two types of ABA synthesis-related genes (*ABA3* and *ABA5*) were identified with similar trends in GS-L, EA-, and GSL–EA-related pathways, with the highest expression observed in the GSL–EA (Figure 6E).

### 2.7. Influence of GSL and EA Priming on Seed Germination-Related Traits under Drought Stress Conditions

**Figure 7:**
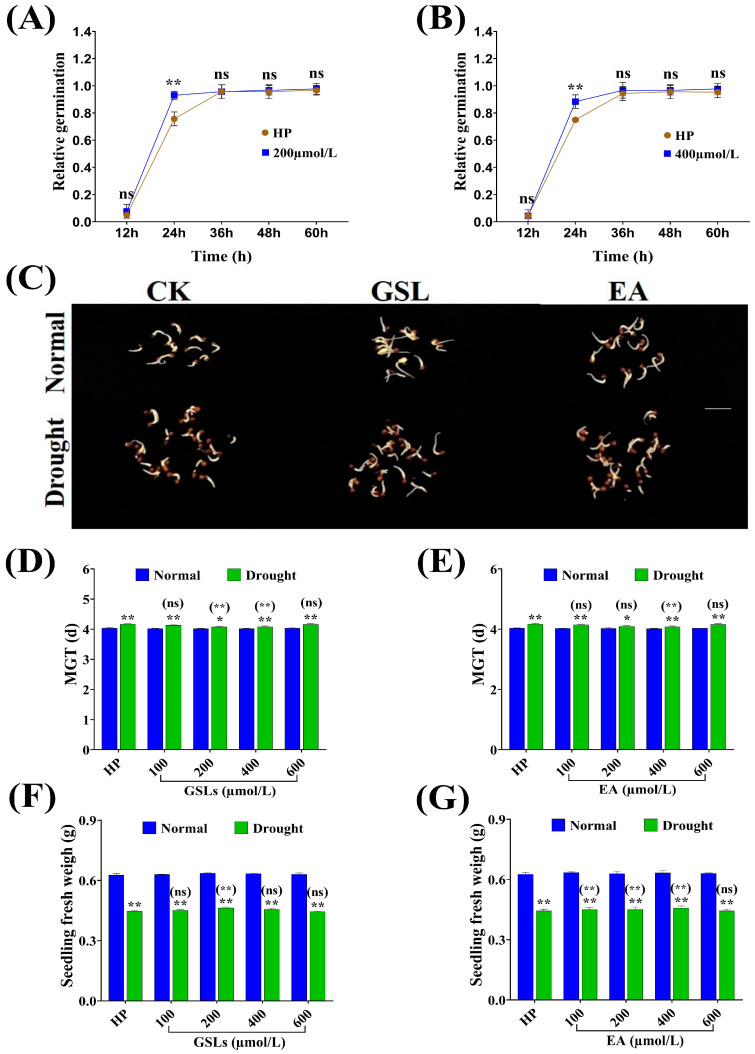
The influence of GSL and EA priming on (**A**,**B**) relative germination%, (**C**) seed germination phenomenon at 24 h of seed imbibition, (**D**,**E**) mean germination time, and (**F**,**G**) seedling fresh weight, respectively, in QY7 (LGLE) under normal and drought stress conditions during the germination and early seedling stage. Bars represent ±SE of three replicates. Asterisks indicate significant differences between drought and normal conditions, while asterisks with parentheses indicate significant differences between HP and different concentrations under drought conditions (ns: non-significant, * *p* < 0.05, ** *p* < 0.01; Student’s *t*-test).

Rapeseed accession (QY7) from the LGLE-type seeds was primed with different concentrations of GSL and EA for more clarification of their roles in seed germination response to drought tolerance. The application of GSL and EA positively affected the G% under drought stress conditions based on their concentrations (Figure S4A–D). Seeds primed with 100, 200, and 400 μmol/L showed significant increases in relative germination of 3.342, 16.61, and 14.43% (GSL) and 1.197, 11.13, and 14.40% (EA), while a decrease of 1.266 and 1.227% at 600 μmol/L of GSL and EA, respectively, versus hydro-primed seeds under drought stress conditions (Figure 7A,B). It was observed that GSL and EA application had a non-significant effect on seed germination and seedling growth under normal conditions. However, under drought stress conditions, priming with GSL (200 μmol/L) and EA (400 μmol/L) significantly promoted seed germination and seedling growth in QY7 from the LGLE-type group (Figure 7C).

On the other hand, versus hydro-primed seeds, mean germination time was decreased by 0.034, 0.092, 0.085, and 0.017 (GSL) and 0.023, 0.076, 0.084, and 0.015 (EA) with priming of 100, 200, 400, and 600 μmol/L of GSL and EA, respectively, under drought stress conditions (Figure 7D,E). Additionally, priming with 100, 200, 400, and 600 μmol/L of GSL and EA significantly increased seedling weight by 3.037, 7.419, 5.118, and 2.238% (GSL), while 1.121, 4.548, 7.812, and 3.557% (EA), respectively, compared to hydro-priming under drought stress conditions (Figure 7F,G). Conclusively, GSL and EA application in LGLE-type seeds was found to be an effective method to enhance seed germination and seedling growth under stress conditions.

### 2.8. Differentail Gene Expression with GSL and EA Priming under Drought Stress Conditions

**Figure 8:**
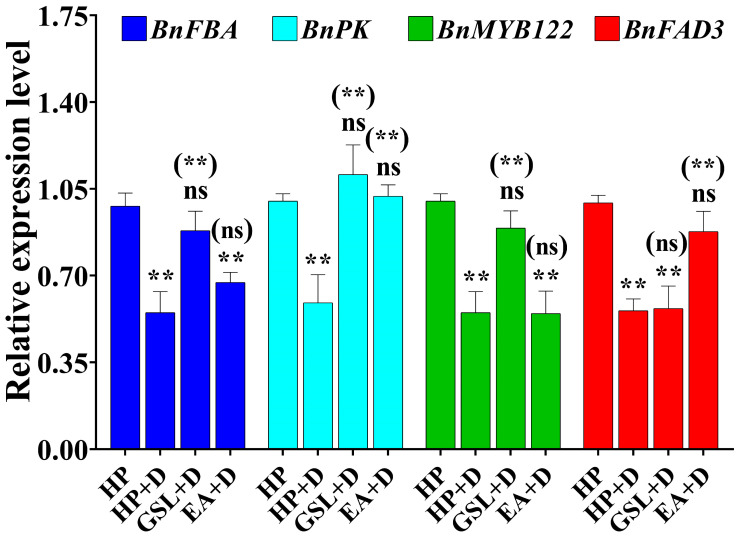
The influence of seed priming via 200 μmol/L of GSL and 400 μmol/L of EA on the relative expression gene in QY7 (LGLE) at 24 h of seed imbibition under drought stress conditions. Bars represent ±SE of three replicates. Asterisks indicate significant differences between hydro-priming (HP) under normal conditions and treatment under drought conditions, while asterisks with parentheses indicate significant differences between HP and other treatments under drought conditions (ns: non-significant, ** *p* < 0.01; Student’s *t*-test).

In the current study, the effects of GSL and EA priming on gene expression were investigated under drought stress conditions using 200 μmol/L of GSL and 400 μmol/L of EA, which showed significant effects on seed germination initiation, particularly at 24 h of seed imbibition (Figure 7C). The expression levels of *BnFBA*, *BnPK*, *BnMYB122*, and *BnFAB3* genes were significantly changed with GSL and EA priming application of QY7 seeds under drought stress conditions. Moreover, *BnFBA* and *BnPK* expression levels were increased by 46.71 and 37.55% (GSL) and 42.15 and 18.14% (EA) versus untreated seeds, respectively, under drought stress conditions (Figure 8). Furthermore, the expression level of the gene encoding myrosinase enzyme (*MYB122*) was significantly increased by 38.25% with GSL exogenous application, while it showed a non-significant increase with EA exogenous application, versus untreated seeds under drought stress conditions. Meanwhile, the *BnFAB3* gene was significantly upregulated by 36.39% with EA seed priming, while it showed a non-significant change with GSL priming treatment, versus untreated seeds under drought stress conditions (Figure 8). Our results indicated that GSL and EA play a vital role in seed germination through improving stress-responsive genes related to glycolysis (*BnFBA*, *BnPK*), GSL synthesis (*MYB122*) and membrane fluidity (*FAD3*) under drought stress conditions.

## 3. Discussion

### 3.1. Different GSL and EA Contents Influenced the Seed Germination-Related Traits under Drought Stress

Seed germination is indeed a critical stage in the life cycle of plants, and it is highly sensitive to climate change, particularly drought [35]. Drought stress significantly affected the physiochemical metabolite-related traits in seeds, leading to reduced water absorption and subsequently affecting seed germination potential [36]. However, a higher G% and shorter MGT of seeds exhibited more robust viability and higher seedling rates under stress conditions [37]. Our study revealed that HGHE, HGLE, and LGHE had a higher G% with shorter MGT, especially at 24 h versus LGLE, which aligned with glucose metabolism trends, indicating that it is crucial for supplying energy and metabolites during the early seed germination response to drought stress [38]. Furthermore, the global expansion of rapeseed seed quality is dependent on seed GSL and EA contents [29]. Under stress conditions, GSL is involved in osmotic adjustment and signaling process [39,40]; besides, EA is assembled and stored as TAGs, which degrade into fatty acids that provide energy for seed germination [41], thus playing a vital role during seed germination. Taken together, these findings suggest that the impact of drought stress on seed germination is largely influenced by the respective GSL and EA contents of rapeseed seeds.

### 3.2. Different GSL and EA Contents Modulated the Physiochemical Indices in Response to Drought Stress

During seed germination, the decomposition of sugar, protein, and oil not only provides energy but also participates in osmoregulation [42,43]. However, drought induces impediments in their mobilization that is attributed to germination inhibition through decreasing energy provisions and osmoregulatory capacity [38,44]. Under drought stress, dynamic changes in sugar, protein, and lipid contents in the four seed types revealed that variations in storage compound mobilization primarily occurred in the glucose degradation pathway between 24-60 h of seed imbibition, where the HGHE demonstrated the least decline in sugar content compared to LGLE. This difference appears notably to influence both energy metabolism and osmoregulatory processes [45,46]. Seed GSL can be decomposed by myrosinase enzymes to produce glucose and sulfate [47,48], which slower the carbohydrate reduction, ensuring a consistent energy source and osmotic protection, thereby maintaining electrochemical gradient stability during the germination under drought stress [49]. Moreover, the degradation of GSL and EA through myrosinase and lipase activity was found to be higher in the HGHE seeds compared to LGLE under drought stress conditions.

Similarly, EA acts as a buffer and osmotic fluid, and its catabolism contributes to increased sugar content, preserving energy metabolism and osmoregulation under drought stress [50]. This may be attributed to a difference in the degradation process caused by the C18:1 structure of EA in the seeds [51]. Among the four studied seed types, drought stress differentially delayed the initiation time and content variation in protein and oil degradation. In the HGHE seeds compared to the other types, especially LGLE, protein reduction was higher than oil and lower than sugar, suggesting that proteins and lipids were primarily consumed during the seedling establishment phase following seed germination [52]. Moreover, FBA and PFK are important enzymes involved in sugar metabolism, which convert 6C sugars into key components of 3C sugars and participate in plant drought regulation [53]. Herein, FBA and PFK contents were increased in the HGHE seeds compared to the other types, especially LGLE, indicating the essential role of GSL and EA under drought stress conditions.

During the germination process, main reserve organs (OBs and PBs) gradually varied in high- and low-oil-content varieties [19], as well as the relation between oil content and EA and GSL [54]. Under drought stress, OB arrangement becomes looser and smaller in size and sectional area, as well as fewer in number in the intercellular space [55]. Our results showed that HGHE had a lower protein-to-oil body area ratio versus other studied successions under drought stress conditions, which enhanced the seed germination rate. Moreover, the void area between PBs and OBs was absent under drought stress conditions in all the studied groups. Drought stress disrupts the normal transportation of solutes, causes electron leakage, and triggers the production of ROS, which creates oxidative injury [56]. Our findings demonstrated a significant increase in POD and CAT activities during germination under drought stress versus normal conditions, particularly in HGHE-type seeds, promoting ROS scavenging, thereby attenuating oxidative cellular damage during adverse conditions [57,58].

Plant hormones are pivotal in regulating plant growth and adaptation to environmental changes [59,60], and their levels can vary during germination among different seed types [18]. ABA and GA are crucial hormones regulating seed germination, and a high GA/ABA ratio is instrumental in transitioning from seed dormancy to germination; however, higher ABA inhibited GA levels and seed germination [61]. Herein, the HGHE demonstrated a higher ABA and slightly higher GA contents that increased the GA/ABA ratio, which was conducive to rapid germination under drought stress compared to other seed types [62]. The drought tolerance of high-GSL and -EA seeds is correlated with ABA metabolism or ABA sensitivity of the seeds [63]. Moreover, HGHE exhibited a significantly higher SA content under drought stress compared to other seed types, especially LGLE, which was beneficial for the improvement of osmoregulation [64]. Additionally, SA improves seed germination and seedling growth-related traits by elevating water absorption, antioxidant activity, and reserve mobilizing enzyme activities, thereby enhancing drought tolerance [65]. GSL content is regulated by auxin-sensitive Aux/IAA repressor proteins through transcriptional cascade reactions, which improves drought stress tolerance [66]. Previous studies have shown the positive correlation between SA, ABA and GSL production in *Brassica alboglabra* and *Brassica rapa* [67,68].

### 3.3. Transcriptional Regulation of GSL- and EA-Related Drought Stress-Responsive Pathways during theSeed Germination

In our investigation, GO enrichment analysis showed higher variations in transcription regulator activity and DNA-binding transcription factors, followed by ATP and microtubule motor activity in GSL–EA-related pathways. The KEGG analysis identified three categories, cellular processes, environmental information processing, and metabolism, with differences in gene participation observed in each category between GSL–EA-related pathways. Notably, peroxisome enrichment was highlighted in the cellular processes category, which is essential for enhancing plant sensitivity to ROS under drought stress conditions [69]. Meanwhile, environmental information was focused predominantly on hormonal signaling that plays a crucial role in regulating seed germination, besides the interactions between hormones collectively affect plant development under stress conditions [26,70]. ABA and GA have a relation with GSL and EA contents during the germination process, and our results showed variations in the genes encoding ABA and GA, especially in HGHE versus other groups, under drought stress conditions at 24 h of seed germination. The gene ABSCISIC ACID-INSENSITIVE 5 (*ABI5*), involved in ABA signaling, including PYR/PYL/RCAR receptors, PP2C phosphatases, and SnRK2 kinases, via regulating genes expression that contain the ABSCISIC ACID RESPONSE ELEMENT (ABRE) motif [71]. GA promoted seed germination by releasing seed dormancy, mobilizing seed reserves through stimulating hydrolase production [72], and regulating genes in the GA signaling pathway, including *SLY1* and *GID1* [13].

In terms of metabolism, variances were primarily seen in photosynthesis-, glycolysis-, and fatty acid-related metabolism pathways as well as cyanoamino acid, α-linolenic acid, alanine, aspartic acid, and glutamate metabolism, which play a vital role during the germination process under drought stress [73]. Metabolites have essential roles in ROS scavenging caused by oxidative stress [74], working as antioxidants and cell wall-strengthening compounds that decrease lipid peroxidation upon drought stress conditions [75]. Furthermore, metabolites promote osmotic adjustment by improving TCA cycle and glycolysis to accelerate energy production and modulate the glutamic acid-mediated proline biosynthesis pathway [76]. Glycolysis is a metabolic process that converts glucose to pyruvate through enzymatic catalysis, which provides energy for cells and sugar metabolism, thus helping cells adapt to stress [77]. Furthermore, glycolytic enzymes, glycolytic rate, and energy production are the main determinants of successful seed germination [78]. Herein, genes encoding key enzymes in glycolysis, such as PFK and PK, were upregulated in germinated seeds, especially in HGHE, which is supported by Bellieny-Rabelo, et al. [79].

### 3.4. GSL and EA Priming Enhanced Drought Tolerance during the Seed Germination of LGLE-Type Seeds

Based on the aforementioned results, differential drought stress response capabilities were found among four rapeseed types, HGHE exhibited the highest tolerance, suggesting a pivotal role for GSL and EA in promoting seed drought tolerance. Additionally, seed exogenous application has been found to be an effective method for enhancing germination stress tolerance [80]. Seeds treated with glucose mitigate organelle damage, including chloroplast decay, membrane damage, and cell death, resulting in a notable increase in stress tolerance [81]. Our results showed that seed priming with 200 μmol/L of GSL or 400 μmol/L of EA significantly improved the G% of QY7 (LGLE) under drought stress conditions. Therefore, GSL and EA can be considered as promising agents for enhancing the drought tolerance of LGLE seeds during the germination stage. Additionally, a previous study reported that priming with GSL and EA improved stress tolerance at the rapeseed seedling stage [82].

Moreover, FBA and PK are involved in glycolysis to break down glucose and reduce the intracellular sugar content, increasing the water potential that regulates the intracellular osmotic pressure, and improving drought tolerance in rapeseed [83]. Our results indicated that the expression levels of *BnFBA1* and *BnPK* genes were increased with GSL versus EA application under drought stress conditions, indicating that *BnFBA* and *BnPK* are stress-responsive genes. The MYB family is the largest class of transcription factors and is involved in regulating secondary metabolism, signal transduction, and stress response [84]. Among them, R2R3-MYB transcription factors positively regulate GSL synthesis-related genes, including *MYB122* that regulating indole GSL synthesis [85], which was increased with GSL application, indicating its involvement in the process of GSL-related response to drought stress.

The degree of FA desaturation is related to the response of plants to various abiotic stresses; moreover, *FAD3* regulates the ratio of total linolenic acid to linoleic acid, which maintains membrane fluidity and increases drought tolerance [86]. Our results showed increased expression level of *FAD3* in LGLE seeds, indicating that GSL and EA play a vital role in seed germination under drought stress conditions.

## 4. Materials and Methods

### 4.1. Plant Materials

A total of 114 *B. napus* accessions were divided into four groups based on different concentrations of glucosinolate (GSL) and erucic acid (EA)—high GSL and high EA (HGHE), high GSL and low EA (HGLE), low GSL and high EA (LGHE), and low GSL and low EA (LGLE)—according to [87,88], which were used in the current study (Table S1).

For transcriptome analysis, three accessions were selected from each group, Qianyou331, Caoyou 2, and Ganyou 5 (HGHE); 7191, Major, and Niklas (HGLE); B262, Huyou 16, and Cubs root (LGHE); and Zhongshuang11, Qinyou7, and Huyou12 (LGLE), at 24 h of seed imbibition under normal and drought stress conditions.

### 4.2. Seed Germination

Uniform-sized seeds (100) were subjected to germination in polyethylene boxes (12 × 12 × 6 cm), and each box containing three sterilized filter paper, which were moistened with either 15 mL of dH_2_O as a control or 15% PEG_6000_ as a drought treatment. The experiments were conducted with three replications and three biological replicates. Germination was conducted at a temperature regime of 25/20 °C, with a 16 h light/8 h dark cycle. Germination % (G%) was determined at time intervals of 12, 24, 36, 48, and 60 h using the radicle emergence through the seed coat as the criterion, calculated as follows:

(1) $$\mathrm{Germination}\;\%\left(G\%\right)=\frac{\mathrm{Number}\;\mathrm{of}\;\mathrm{germinated}\;\mathrm{seeds}}{\mathrm{Total}\;\mathrm{number}\;\mathrm{of}\;\mathrm{seeds}}\times 100$$

(2) $$\mathrm{Mean}\;\mathrm{germination}\;\mathrm{time}\;\left(\mathrm{MGT}\right)=\frac{\sum \left(\mathrm{Gt}\times \mathrm{Dt}\right)}{\sum \mathrm{Gt}}$$

where

Gt: number of germinated seeds on day t
Dt: day after sowing

(3) $$\mathrm{Relative}\;\mathrm{germination}=\frac{\mathrm{GP}\;\mathrm{under}\;\mathrm{drought}\;\mathrm{stress}}{\mathrm{GP}\;\mathrm{under}\;\mathrm{normal}\;\mathrm{conditions}}$$

(4) $$\mathrm{Coefficient}\;\mathrm{of}\;\mathrm{variance}\;\left(\mathrm{CV}\right)=\frac{\mathrm{Standard}\;\mathrm{deviation}\;\left(\mathrm{SD}\right)}{\mathrm{Average}\;\mathrm{value}}\times 100$$

Based on the germination analysis at different time points, four representative accessions were selected from the experiments above, HGHE (Caoyou2), HGLE (7191), LGHE (Cubs root), and LGLE (Qinyou7), to continue further investigation.

### 4.3. Transmission Electron Microscopy (TEM) Analysis

An ultrastructural study of the four accessions seeds was carried out using TEM, and sample preparation, observation, and data acquisition were conducted following the method mentioned by El-Badri, et al. [89]. Ultra-thin sample sections were observed using an H7650 TEM and analyzed using the ImageJ (v1.8.0) software.

### 4.4. Oil Content Determination

Seed samples (1 g) were dried at 105 °C for 8 h and re-weighed after cooling, followed by overnight soaking in anhydrous ether. Samples were distilled at 60 °C using a foil extractor until the color of the ether became clear. Then samples were dried at 105 °C for 8 h, followed by cooling and weighing [90].

### 4.5. Total Soluble Sugar and Protein Contents

The total soluble sugar content of rapeseed seeds was determined using the classical anthrone sulfuric acid method [91]. While total soluble protein content was measured using Coomassie brilliant blue G-250 method [19]. The absorbance values were measured at 620 and 595 nm, respectively.

### 4.6. Myrosinase, Lipase, FBA and PFK Activity Determination

A total of 500 mg of seed sample was digested in ethanesulfonic acid (MES) buffer (pH 6.0) at 0 °C, then the mixture was incubated at 25 °C for 5 min, and the supernatants were collected after centrifugation for myrosinase activity measurements [92]. Samples were extracted using phosphoric acid buffer (pH 7.8), and then lipase enzyme activity was measured using assay kits (A054-2) from the Nanjing Jiancheng Bioengineering Institute, Nanjing, China. Meanwhile, fructose-bisphosphate aldolase (FBA) and 6-phosphofructokinase I (PFK) activities were determined at 340 nm using commercial assay kits (Solarbio, Beijing, China; BC2275 and BC0535, respectively) [93,94].

### 4.7. Determination of MDA Content and Antioxidant Enzyme Activity

Trichloroacetic acid (TCA) was used to detect MDA content in the seed samples, and OD values were measured at 532, 600, and 450 nm according to the method mentioned by [95]. Additionally, samples were extracted using phosphoric acid buffer (pH 7.8) to measure the antioxidant enzyme activity using assay kits (A001-1 and A084-3) from the Nanjing Jiancheng Bioengineering Institute, Nanjing, China.

### 4.8. Endogenous Hormones Determination

To determine the content of abscisic acid (ABA), gibberellic acid (GA), salicylic acid (SA), and indole acetic acid (IAA) hormones, 20 mg of freeze-dried seed samples were mixed with cold extraction buffer (methanol:water:acetic acid, 80:19:1, *v*/*v*/*v*) followed by centrifugation then supernatant were collected for liquid chromatography. The analysis was conducted using an ALTIS triple quadrupole liquid chromatography–mass spectrometer, with quantitative data acquisition and processing performed using the Thermo Trace Finder 3.2 software [96].

### 4.9. Transcriptome Sequencing and Differential Gene Analysis

#### 4.9.1. Sample Preparation and RNA Extraction

Germinated seed samples (24 h) of three accessions from each group were used to extract the total RNA using the Easystep^®^ Super Total RNA extraction kit (Shanghai Promag Bioproducts Co., Ltd., Shanghai, China). The integrity and quantification of total RNA were determined using the 2100 Bioanalyzer (Agilent Technologies, Inc., Santa Clara, CA, USA) and NanoDrop (Thermo Scientific, Wilmington, MA, USA), respectively [97].

#### 4.9.2. RNA-Seq Analysis

RNA-seq libraries were constructed according to the user manual (Illumina, http://www.illumina.com/ (accessed on 23 August 2023)) and sequenced to produce 150 bp paired-end reads. FastQC software (Version: 0.11.9) was used to evaluate the quality of the raw data, and Trimmomatic (Version: 0.39) was used to remove low-quality reads and linker sequences to obtain clean data. The clean data were compared with Zhong Shuang 11 (ZS11) as a reference genome (http://cbi.hzau.edu.cn/bnapus/ (accessed on 28 August 2023)) using Hisat2 software (Version: 2.1.0). The number of the comparison on each gene was calculated using the FeatureCounts tool in the subread software (Version: 2.0.1), and only the compared reads were counted.

#### 4.9.3. Analysis of Differentially Expressed Genes (DEGs)

Libraries were prepared and sequenced using DNBSEQ technology at Paysenno Biotechnology Co., Ltd. (Shanghai, China). Differential expression across the groups was analyzed using DESeq (1.30.0) software. The *p* values were adjusted using the Benjamini–Hochberg procedure to manage the false discovery rate, and the differential gene expression screening conditions were as follows: Padj ≤ 0.05, |Fold change| ≥ 1, and the average gene expression level FPKM ≥ 1.

#### 4.9.4. Gene Functional Enrichment Analysis

Gene ontology (GO) and Kyoto Encyclopedia of Genes and Genomes (KEGG) enrichment analyses were conducted using the clustering Profiler (3.4.4) software. Concurrently, the phyper function in the R (v3.1.1) software was utilized for further enrichment analysis. After calculating the q value, FDR correction was applied and functions with a q value ≤ 0.05 were significantly enriched.

### 4.10. Exogenous Application of GSL and EA

For more clarification based on our results, different concentrations of GSL and EA were used for further experiments. Rapeseed accession from the LGLE group (QY7) seeds were primed with different concentrations of GSL and EA: 0, 100, 200, 400, and 600 μmol/L. After the treatment, seed germination assays were performed under normal and drought conditions to evaluate G% and MGT.

### 4.11. RNA Extraction, cDNA Synthesis, and Quantitative RT-PCR

The cDNA was synthesized from the total RNA of unprimed and primed seeds using TransScript One-Step gDNA Removal and cDNA Synthesis SuperMix kits (TransGen, Beijing, China). Quantitative real-time PCR was performed using TransStart Tip Green qPCR SuperMix (TransGen, Beijing, China) with the Roche LightCycler 480 thermal cycler instrument, 384-well (Roche). Relative expression values were calculated using the 2^−ΔΔCt^ method according to Lou, et al. [98]. *ACT7* was used as a reference gene, and the primer sequences are listed in Table S2. Three biological replicates with three technical replicates were performed for each sample.

### 4.12. Data Analysis

Data were subjected to analysis of variance (ANOVA) using SPSS 17.0 software (Inc., Chicago, IL, USA). Student’s *t*-test and Duncan differential significance analysis were used to analyze the significance differences between the treatments. Graphical presentations were carried out using the Origin 9.0 software (OriginLab Corp, Northampton, MA, USA).

## 5. Conclusions

The current study found that seed GSL and EA contents play a vital role in alleviating drought stress during seed germination in rapeseed. The HGHE group, compared to LGLE, exhibited superior mobilization rates of sugars, proteins, and lipids, as well as ultrastructural changes in OBs and PBs, providing more energy for seed germination. Moreover, the enzymatic system was improved in HGHE with a decreasing MDA content, which reduced oxidative stress. During the seed germination process, hormonal regulation was significantly positively varied in HGHE seeds versus other types, which helps protect seed germination under drought stress conditions. Transcriptome analysis revealed distinct transcriptional regulatory differences among the four seed types during germination in response to drought stress. LGHE showed enhanced glucose and lipid metabolism, while HGLE excelled in sugar metabolism and ROS mitigation. The HGHE-type seeds had strengths in glucose and hormone metabolism as well as ROS scavenging. Moreover, the exogenous application of GSL and EA, at 200 and 400 μmol/L, respectively, significantly improved G%, MGT, and seedling fresh weight, as well as regulating stress- and fatty acid-responsive genes in QY7 (LGLE) under drought stress conditions. Therefore, GSL and EA can be considered as promising agents for enhancing the drought tolerance of LGLE seeds during the germination stage.

## Data Availability

Data is contained within the article.

## Supplementary Materials

The following supporting information can be downloaded at: https://www.mdpi.com/article/10.3390/ijms25063308/s1.
