# Supplementary material for: Morpho-Physiochemical Indices and Transcriptome Analysis Reveal the Role of Glucosinolate and Erucic Acid in Response to Drought Stress during Seed Germination of Rapeseed
Source: Int J Mol Sci. 2024 Mar 14;25(6):3308. doi: 10.3390/ijms25063308 (PMC10970144; doi:10.3390/ijms25063308)
Supplement: Supplementary file 1 [file ijms-25-03308-s001.zip › ijms-2891870-supplementary.pdf]

Table S1. Seed quality and germination parameters of 114 rapeseed accessions.

Group	No.	Name	PC	OC	EA	GSL	C18:3	C18:2	C18:1	Source
HGHE (++)	1	Ganyou5	26.38	39.25	21.07	75.79	8.326	9.681	40.83	Hubei (China)
	2	Kelina	21.28	44.9	36.29	104.2	9.535	9.222	20.15	Chongqing (China)
	3	Qianyou331	18.92	48.77	38.73	90.54	7.176	10.44	19.21	Guizhou (China)
	4	Enyou73-1-2	24.38	44.40	34.24	114.2	8.944	10.59	21.83	Chongqing (China)
	5	Niuerduo	24.24	42.06	29.03	129.4	9.284	9.885	28.42	Chongqing (China)
	6	880101	20.54	44.40	24.61	94.43	8.161	11.22	34.38	Chongqing (China)
	7	Swu40	21.23	43.15	21.26	110.8	8.543	11.27	39.73	Chongqing (China)
	8	96063	21.38	43.25	32.49	116.9	9.007	9.520	26.10	Hubei (China)
	9	1111	21.10	44.50	34.14	118.2	8.501	8.594	25.39	Hubei (China)
	10	Huashuang2	21.17	43.68	17.67	88.01	8.357	15.43	39.92	Hubei (China)
	11	Rucabo	21.61	45.19	34.34	104.1	7.725	8.676	24.73	German
	12	P158	22.67	39.85	31.97	106.8	8.664	13.09	22.35	Shanxi (China)
	13	03I32B	20.07	45.60	26.75	96.8	7.272	12.13	33.26	Gansu (China)
	14	Nakaee [illegible]	23.88	44.81	28.61	108.3	8.331	11.53	27.58	Japan
	15	Cat.No.117	24.54	41.52	33.59	122.8	8.401	10.95	21.63	Union of Soviet
	16	90750	20.96	42.34	30.50	90.77	8.285	11.97	25.86	Chongqing (China)
	17	Norin43	21.37	40.11	17.59	89.10	8.284	14.60	43.35	Japan
	18	Youyan2	20.92	44.29	33.65	104.1	8.830	11.07	21.23	Guizhou (China)
	19	Aijiazao	20.05	45.98	33.24	106.0	7.910	11.28	21.31	Sichuan (China)
	20	Swu57	22.36	46.73	27.53	110.5	8.321	13.20	27.04	Chongqing (China)
	21	Huyou15	20.84	45.40	29.37	68.35	7.256	11.50	28.63	Shanghai (China)
	22	Weilong88	21.48	43.12	21.73	110.5	7.690	11.29	39.24	Hubei (China)
	23	Hector	23.22	44.03	34.78	114.9	9.179	10.58	18.56	Hubei (China)
	24	Shengliyoucai	25.24	39.89	25.96	105.4	9.001	10.59	32.09	Japan
	25	Huayou4	22.18	44.06	21.04	113.6	9.319	12.33	37.03	Hubei (China)
	26	Shengliqinggeng	21.57	41.86	28.41	91.83	8.538	13.25	27.54	Shanghai (China)
	27	aijishengli	21.13	42.69	33.86	88.98	7.429	9.88	25.95	Shanghai (China)
	28	Caojingshengli	20.86	44.15	33.85	91.11	8.967	10.42	20.54	Shanghai (China)
	29	Huyou3	23.09	43.61	30.69	118.8	9.204	11.19	26.36	Shanghai (China)
	30	Shaoyeqing	18.80	45.19	31.29	95.24	8.423	11.45	24.09	Shanghai (China)
	31	Caoyou2	20.69	45.27	36.91	110.3	8.282	11.58	17.28	Shanghai (China)
	32	Fengding240	21.15	48.17	30.19	88.07	7.094	9.763	29.89	Jiangsu (China)
	33	Quanziyoucai	20.40	46.91	29.15	100.4	7.972	10.48	29.98	Jiangsu (China)
	34	huaiyou6	24.80	43.09	31.88	113.6	8.681	10.33	24.94	Japan
	35	Guangde138	19.59	48.42	38.71	106.5	7.447	9.96	18.17	Anhui (China)
	36	Guangde8104	22.59	45.09	31.44	121.2	8.167	10.04	28.84	Anhui (China)
	37	Tonglinghuaye	20.50	47.56	38.29	109.4	7.218	8.45	20.83	Anhui (China)
	38	Chu610	21.73	47.76	32.14	128.1	8.862	10.02	26.56	Anhui (China)
LGLE (00)	1	Swu47	19.12	43.23	1<	22.00	6.935	17.97	69.63	Chongqing (China)
	2	Swu92	19.97	44.05	1<	27.62	7.333	17.90	69.31	Chongqing (China)
	3	Xiangyou11	18.78	45.66	1<	24.68	7.811	15.74	70.02	Hunan (China)
	4	740	19.36	44.06	1<	27.38	7.680	18.11	68.29	Hunan (China)
	5	7022	21.85	41.91	1<	17.68	8.963	18.17	66.77	Hubei (China)
	6	Rr009	20.36	46.27	1<	24.80	7.480	19.53	65.52	Australia
	7	Rr002	20.29	47.22	1<	25.72	7.742	18.01	67.68	Australia
	8	11-P63-3	17.84	45.37	1<	19.87	7.254	16.99	66.31	Hubei (China)
	9	23651	19.26	43.82	1<	26.61	7.463	15.40	68.74	Hubei (China)
	10	Huashuang5	18.35	49.74	1<	28.49	7.857	16.53	64.36	Hubei (China)

	11	Huashuang4	17.41	48.90	1<	29.94	6.959	16.52	69.14	Hubei (China)
	12	Jia904	18.40	46.05	1<	29.30	7.217	16.96	68.32	Hubei (China)
	13	Jiapf190	18.14	44.39	1<	28.68	7.410	19.10	66.85	Hubei (China)
	14	Jia951	19.59	43.48	1<	27.42	7.556	18.42	66.17	Hubei (China)
	15	Huyou17	21.16	43.35	1<	20.56	7.938	17.57	67.66	Shanghai (China)
	16	Huyou12	21.24	42.59	1<	22.87	8.168	16.91	67.62	Shanghai (China)
	17	Ningyou12	22.27	37.99	1<	17.30	9.868	17.34	65.49	Jiangsu (China)
	18	Zheyou18	22.11	41.77	1<	26.00	7.176	17.84	66.98	Zhejiang (China)
	19	Zheyou758	23.47	40.77	1<	27.32	7.863	17.36	68.71	Zhejiang (China)
	20	Zheyou21	20.17	46.66	1<	27.51	7.688	15.80	68.47	Zhejiang (China)
	21	Wanyou15	21.82	46.05	1<	24.65	7.862	17.30	66.19	Anhui (China)
	22	Wanyou29	21.40	42.86	1<	22.59	7.383	16.09	69.28	Anhui (China)
	23	Longyou2	18.04	45.25	1<	28.04	7.623	17.74	65.70	Gansu (China)
	24	Swu90	20.01	41.37	1<	16.93	8.706	18.58	64.20	Chongqing (China)
	25	Swu95	19.21	43.92	1<	24.95	6.711	17.20	67.38	Chongqing (China)
	26	Zhongshuang12	19.20	45.70	1<	23.39	7.808	17.13	67.24	Hubei (China)
	27	Fuyou4	18.72	46.51	1<	22.71	7.833	16.64	67.70	Heilongjiang(China)
	28	Zhen3736	19.61	45.57	1<	23.61	8.383	16.56	66.46	Jiangsu (China)
	29	L401	16.52	49.40	1<	27.37	7.578	17.51	65.68	Hubei (China)
	30	Y61	21.26	46.30	1<	30.81	7.448	16.84	67.20	Zhejiang (China)
	31	Zaofeng1	18.26	45.99	1<	27.24	7.135	15.95	68.31	Zhejiang (China)
	32	Huayouza62	28.81	41.18	1.860	29.54	7.945	17.29	62.63	Hubei (China)
	33	Jinzayou158	25.65	43.04	1.650	29.39	6.900	15.34	58.16	
	34	Qinyou7	26.82	42.27	1.028	28.83	8.708	17.50	60.11	Shanxi (China)
	35	Qingyou3	24.20	46.61	1.000	29.31	7.020	15.64	65.51	Chongqing (China)
	36	Xiangzayou518	26.32	46.35	1.000	21.13	8.345	18.73	69.36	Hunan (China)
	37	Zheyou51	23.81	46.98	1.980	27.27	7.990	16.93	64.29	Zhejiang (China)
HGLE (+0)	1	7189	20.44	43.64	1<	61.11	7.228	17.61	67.57	Hubei (China)
	2	7191	19.82	43.08	1<	57.51	7.642	16.18	69.03	Hubei (China)
	3	Major	19.92	42.23	1<	52.55	7.735	19.00	64.41	France
	4	Shilifeng	22.04	41.73	1<	48.63	9.019	17.27	64.24	Jiangsu (China)
	5	Q-247	20.31	45.25	1<	84.37	8.464	16.24	67.71	the USA
	6	11-540	20.28	51.48	1<	50.26	8.361	18.50	65.71	Qinghai (China)
	7	B414	18.32	44.59	1<	71.79	8.481	19.45	64.80	Xinjiang (China)
	8	A148	22.21	42.91	1<	59.49	9.248	17.97	65.23	Sweden
	9	06T9F	20.14	40.30	1<	71.52	9.354	18.06	65.97	Gansu (China)
	10	Swu68	18.91	45.28	1<	62.02	7.389	13.56	72.62	Chongqing (China)
	11	Swu99	20.57	40.71	1<	77.95	6.102	15.09	69.55	Chongqing (China)
	12	Zheshuang3	19.14	46.89	1<	78.15	8.182	17.58	65.23	Zhejiang (China)
	13	Yanyou2	21.76	40.64	1<	52.18	8.353	18.73	63.45	Jiangsu (China)
	14	Cresor	20.69	40.91	1<	71.88	8.314	17.35	63.23	France
	15	L508	20.73	43.98	1<	103.6	9.471	18.22	65.97	Jiangsu (China)
	16	Ningyou8	22.41	41.94	1<	102.1	10.16	18.03	65.14	Jiangsu (China)
	17	Ningyou6	21.30	43.73	1<	102.2	8.316	15.74	68.02	Jiangsu (China)
	18	7037	20.48	42.90	1<	60.35	7.421	17.62	67.40	Hubei (China)
	19	Yangyou5	19.98	42.59	1<	58.58	7.947	16.43	67.96	Jiangsu (China)
	20	Swu49	19.54	44.80	1<	63.60	7.618	16.98	71.39	Chongqing (China)
	21	Huahang901	19.47	48.03	1<	48.67	6.825	15.91	70.18	Hubei (China)
	22	Niklas	18.45	45.04	1<	63.62	7.721	16.06	69.59	Denmark

**LGHE (0+)**	**1**	**Swu53**	**20.66**	**45.59**	**12.33**	**27.58**	**7.287**	**14.82**	**47.22**	**Chongqing (China)**
	**2**	**Swu56**	**19.77**	**45.43**	**6.318**	**20.85**	**7.227**	**14.69**	**58.03**	**Chongqing (China)**
	**3**	**Gy270**	**19.06**	**48.64**	**10.91**	**25.37**	**6.861**	**14.76**	**51.50**	**Shanxi (China)**
	**4**	**Gy284**	**19.19**	**51.54**	**31.63**	**29.81**	**6.217**	**12.00**	**24.19**	**Shanxi (China)**
	**5**	**B262**	**21.28**	**52.09**	**25.16**	**29.43**	**6.457**	**12.18**	**31.14**	**Henan (China)**
	**6**	**Zhenyou5**	**20.03**	**46.26**	**4.439**	**29.46**	**8.597**	**16.96**	**54.68**	**Jiangsu (China)**
	**7**	**L393**	**20.66**	**48.48**	**25.18**	**29.42**	**6.817**	**12.65**	**31.79**	**Shanghai (China)**
	**8**	**Wh-83**	**19.96**	**51.57**	**19.19**	**25.49**	**6.619**	**13.21**	**38.34**	**Hubei (China)**
	**9**	**Cubs Root**	**19.65**	**47.10**	**13.18**	**23.82**	**6.890**	**15.00**	**46.07**	**Korea**
	**10**	**Dd1**	**17.85**	**55.91**	**34.39**	**29.40**	**5.627**	**9.936**	**24.77**	**Gansu (China)**
	**11**	**Wu164**	**17.76**	**56.06**	**37.32**	**28.77**	**6.027**	**9.874**	**14.93**	**Gansu (China)**
	**12**	**Swu90**	**17.38**	**55.96**	**36.07**	**28.78**	**5.565**	**9.862**	**19.30**	**Chongqing (China)**
	**13**	**Wh-55**	**18.16**	**56.07**	**35.10**	**27.47**	**5.443**	**9.755**	**24.15**	**Hubei (China)**
	**14**	**15-6-3424-1**	**18.66**	**55.89**	**33.55**	**28.34**	**5.088**	**9.609**	**27.71**	**Hubei (China)**
	**15**	**15-6-3518-4**	**16.81**	**53.49**	**27.70**	**26.39**	**5.782**	**13.08**	**35.66**	**Hubei (China)**
	**16**	**9Bao22**	**20.06**	**47.35**	**8.179**	**27.19**	**6.442**	**14.77**	**55.18**	**Hubei (China)**
	**17**	**15-6-3004-3**	**20.00**	**50.40**	**20.53**	**28.89**	**6.534**	**13.45**	**38.90**	**Hubei (China)**

Table S2. Primers sequences.

No.	Name	Forward	Reverse
1	***MYB122***	TCCACACCAAACTCTTCTAATC	TCCCGTCATATCTTCTACGTCA
2	***FAD3***	GGCCATTGCCGCCGTGCATTTTGAT	CATGGTTCTGGTGGTGTGTC
1	***FBA***	TACCTGGCATCAAAGTCGACAA	TCG TAGTACTTCTTGCAACGCT
2	***PK***	TCTGATCCTAGTTTCGTACCTT	GGTTACTTGTTTGATTCGGTC
3	***ACT7***	TGGGTTTGCTGGTGACGAT	TGCCTAGGACGACCAACAATACT

Table S3. Correlation coefficient between seed germination and quality related traits.

	RGP	RMGT	PC	OC	EAC	GSLC	LNAC	LAC	OAC
**RGP**	1.00								
**RMGT**	-0.913**	1.00							
**PC**	0.093	-0.044	1.00						
**OC**	0.081	-0.115	-0.520**	1.00					
**EAC**	0.221*	-0.198*	0.135	0.298**	1.00				
**GSLC**	0.286**	-0.217*	0.302**	-0.247**	0.643**	1.00			
**LNAC**	-0.066	0.153	0.484**	-0.675**	-0.055	0.490**	1.00		
**LAC**	-0.209*	0.174	-0.115	-0.286**	-0.938**	-0.633**	0.092	1.00	
**OAC**	-0.205*	0.181	-0.132	-0.281**	-0.993**	-0.641**	0.011	0.914**	1.00

RGP: relative germination percentage at 24 h of seed imbibition; RMGT: relative mean germination time; PC: protein content; OC: oil content; EAC: erucic acid content; GSLC: glucosinolate content; LNAC: linolenic acid content; LAC: linoleic acid content, and OAC: oleic acid content. Significance denoted as * P <0.05 and ** P <0.01.

Table S4. Significance differences of oil, sugar and protein contents among treatments at different time points.

Time point	Oil content (%)				Sugar content (mg/g)				Protein content (mg/g)			
	HGHE	HGLE	LGHE	LGLE	HGHE	HGLE	LGHE	LGLE	HGHE	HGLE	LGHE	LGLE
**6h**	ns	ns (ns)	ns (*)	ns (*)	ns	ns (**)	ns (ns)	ns (ns)	ns	ns (**)	ns (*)	ns (ns)
**12h**	**	ns (*)	ns (*)	ns (**)	ns	ns (**)	ns (ns)	ns (ns)	ns	ns (**)	ns (ns)	ns (ns)
**18h**	*	ns (**)	ns (**)	ns (ns)	ns	ns (**)	** (ns)	ns (ns)	ns	ns (**)	* (ns)	ns (ns)
**24h**	ns	ns (**)	ns (**)	ns (ns)	*	* (**)	* (ns)	** (ns)	**	ns (**)	* (*)	ns (*)
**30h**	*	ns (ns)	ns (ns)	ns (*)	**	* (**)	ns (**)	** (**)	ns	** (**)	ns (ns)	ns (**)
**36h**	**	** (**)	ns (*)	* (ns)	**	** (**)	** (**)	** (**)	**	ns (ns)	* (ns)	ns (**)
**48h**	**	* (**)	ns (ns)	ns (**)	**	ns (ns)	ns (ns)	** (**)	ns	* (**)	ns (**)	** (**)
**60h**	**	* (**)	** (ns)	ns (**)	ns	ns (*)	ns (ns)	** (*)	ns	ns (*)	ns (ns)	ns (*)

Asterisks indicate significant differences between drought and normal conditions, while asterisks with parentheses indicate significant differences between HGHE and other three groups under drought conditions (ns: non-significant, * $P < 0.05$, ** $P < 0.01$; Student's t-test).

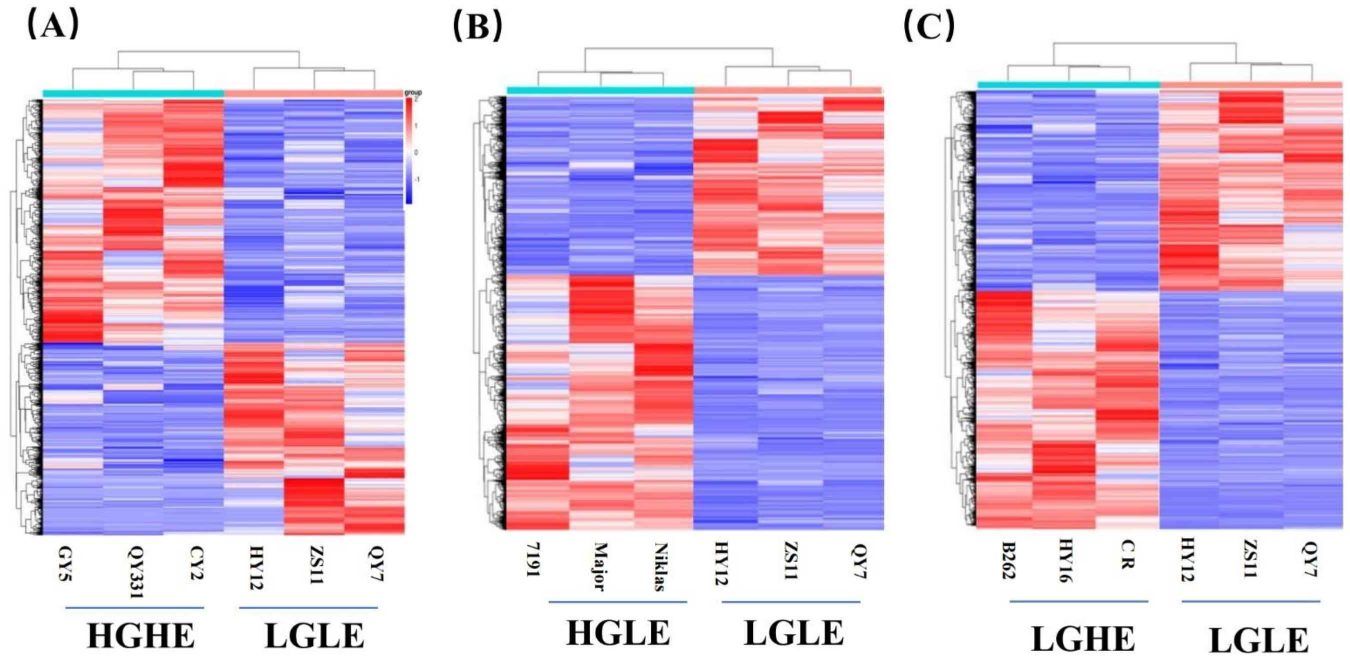

Figure S1. Comparative cluster analysis and heatmap of four seed type groups during seed germination under drought stress conditions.

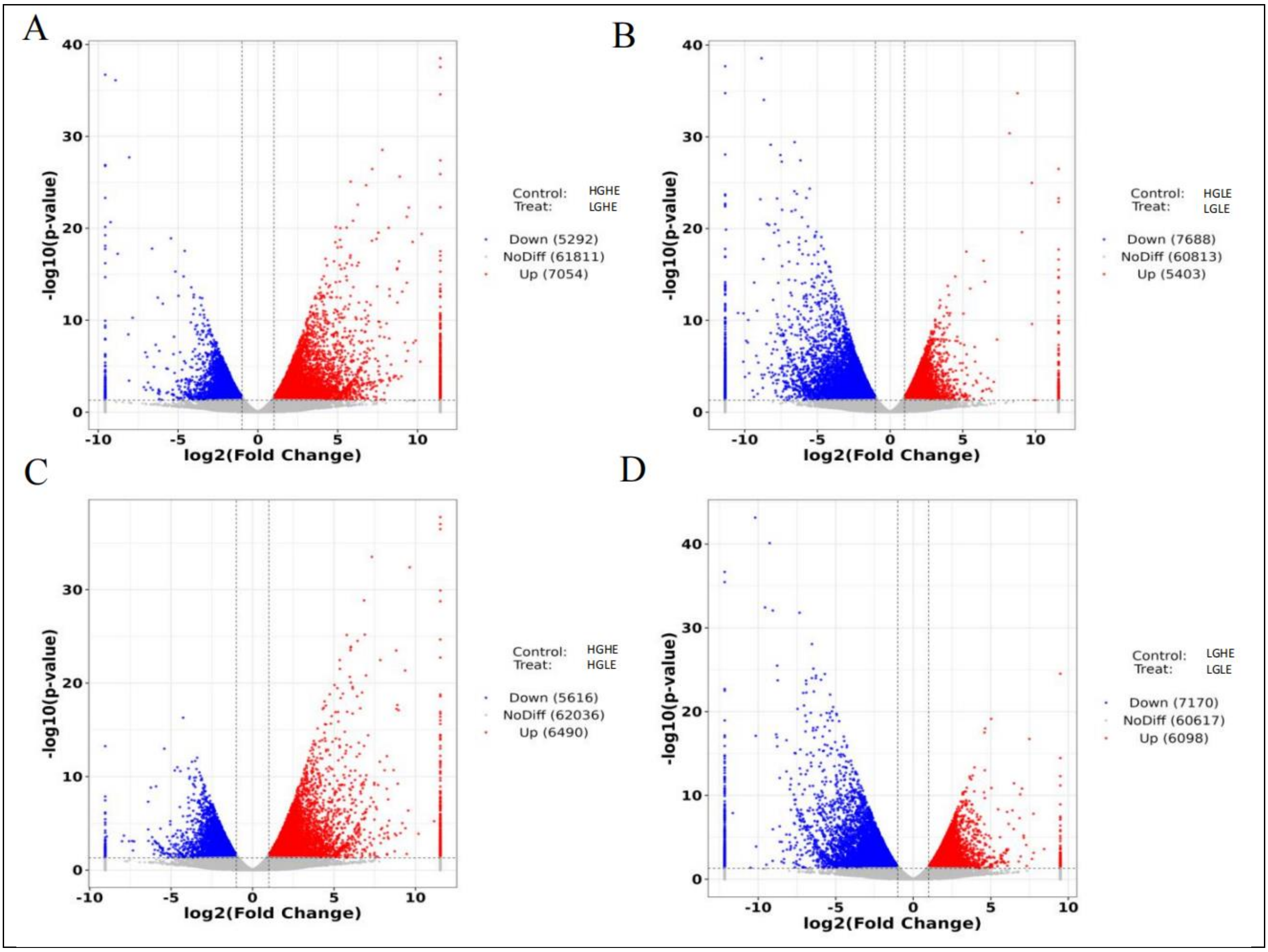

Figure S2. Up- and down-regulated genes of (A) HGHE vs LGHE, (B) HGLE vs LGLE, (C) HGHE vs HGLE and (D) LGHE vs LGLE during seed germination under drought stress conditions.

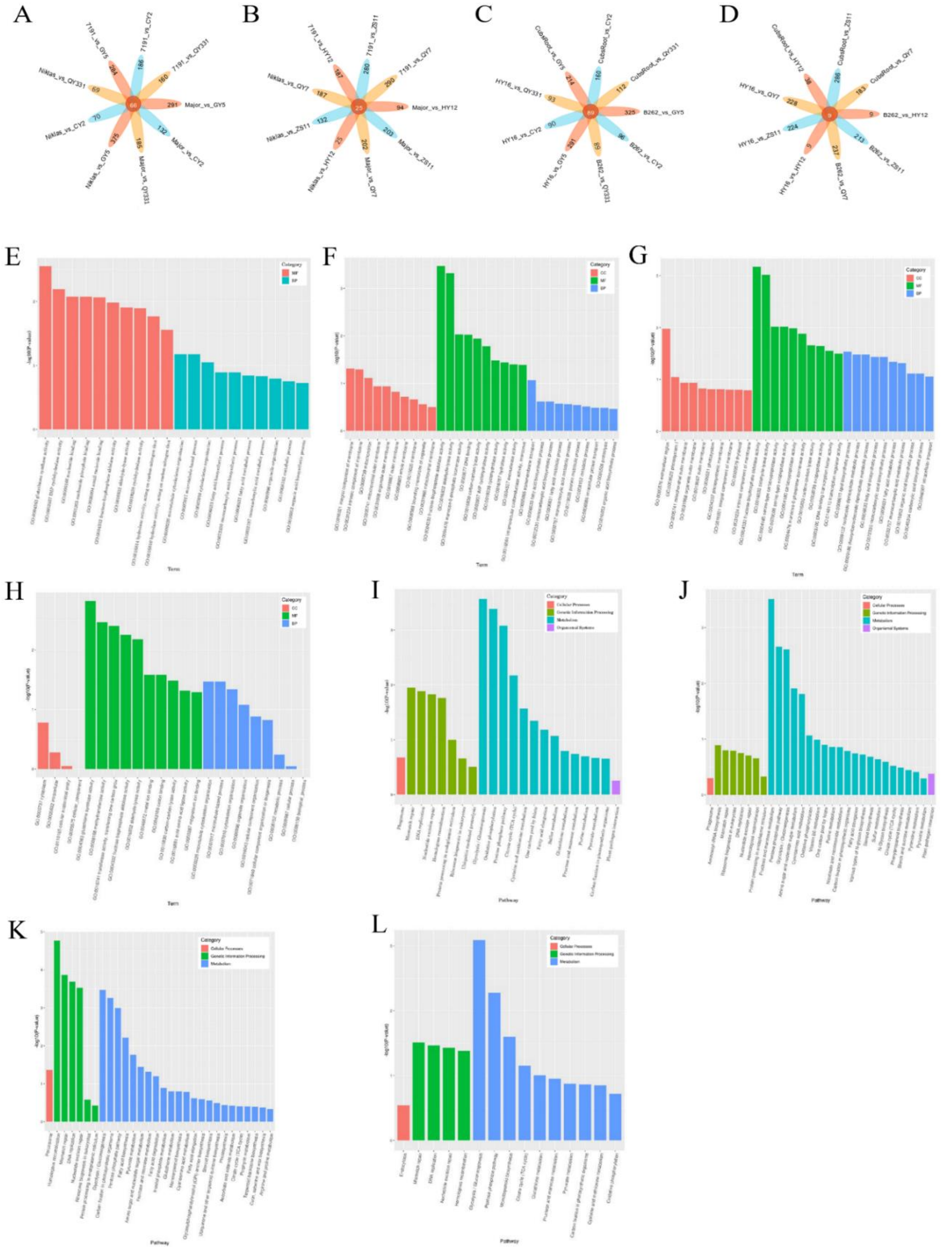

Figure S3. (A-D) Venn diagram, (E-H) gene ontology and (I-L) KEGG enrichment analysis of HGLE with HGHE, HGLE with LGLE, LGHE with HGHE and LGHE with LGLE, respectively.

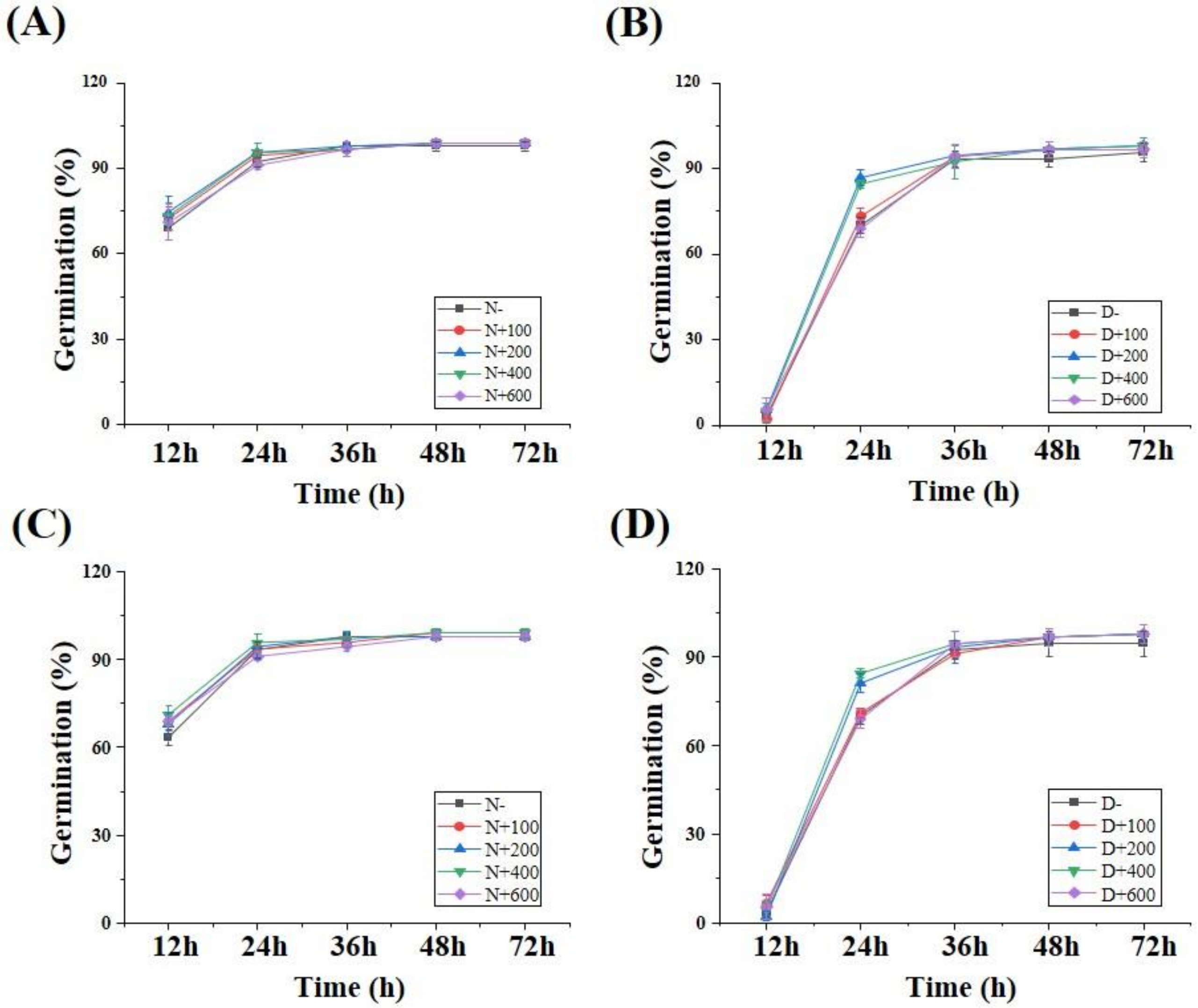

Figure S4. Influence of priming application on germination% via (A and B) GSL (C and D) EA under normal and drought stress conditions, respectively.

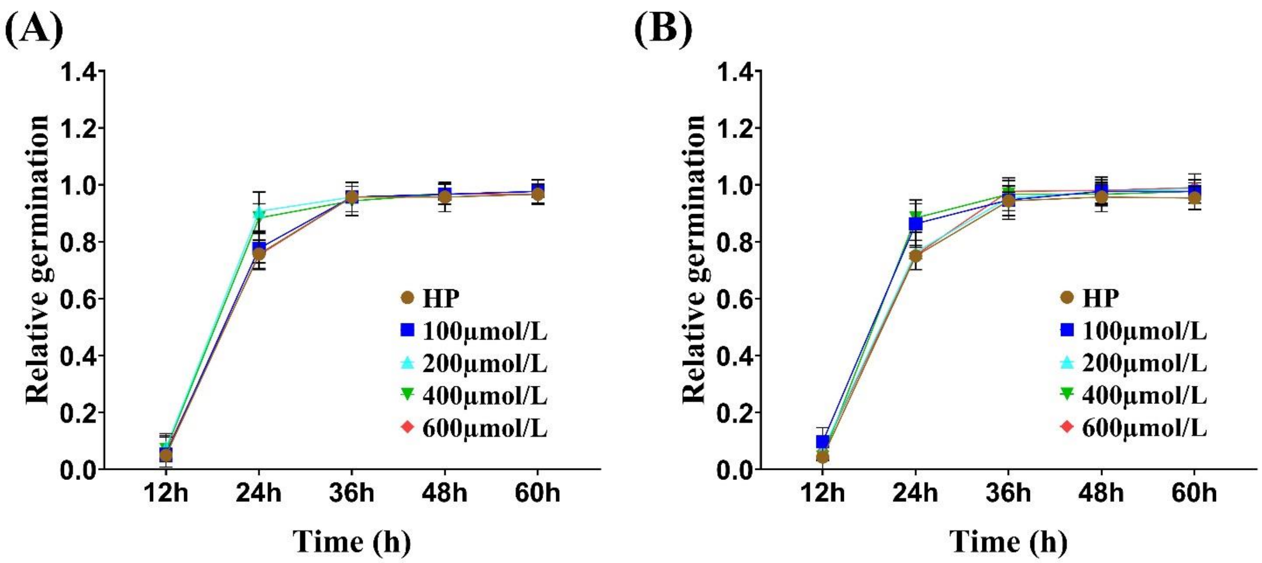

Figure S5. Influence of GSL and EA priming on (A and B) relative germination% under normal and drought stress conditions during the germination and early seedling stage.
